# Development and evaluation of a novel high-throughput image-based fluorescent neutralization test for detection of Zika virus infection

**DOI:** 10.1371/journal.pntd.0006342

**Published:** 2018-03-15

**Authors:** Andrea Cristine Koishi, Andréia Akemi Suzukawa, Camila Zanluca, Daria Elena Camacho, Guillermo Comach, Claudia Nunes Duarte dos Santos

**Affiliations:** 1 Laboratório de Virologia Molecular, Instituto Carlos Chagas (ICC/ Fiocruz- PR), Curitiba, Paraná, Brazil; 2 Laboratorio Regional de Diagnostico e Investigación del Dengue y otras Enfermedades Virales, Instituto de Investigaciones Biomédicas de la Universidad de Carabobo (LARDIDEV/BIOMED-UC), Maracay, Venezuela; Molecular Biology Unit (MBU), INDIA

## Abstract

Zika virus (ZIKV) is an emerging arbovirus belonging to the genus flavivirus that comprises other important public health viruses, such as dengue (DENV) and yellow fever (YFV). In general, ZIKV infection is a self-limiting disease, however cases of Guillain-Barré syndrome and congenital brain abnormalities in newborn infants have been reported. Diagnosing ZIKV infection remains a challenge, as viral RNA detection is only applicable until a few days after the onset of symptoms. After that, serological tests must be applied, and, as expected, high cross-reactivity between ZIKV and other flavivirus serology is observed. Plaque reduction neutralization test (PRNT) is indicated to confirm positive samples for being more specific, however it is laborious intensive and time consuming, representing a major bottleneck for patient diagnosis. To overcome this limitation, we developed a high-throughput image-based fluorescent neutralization test for ZIKV infection by serological detection. Using 226 human specimens, we showed that the new test presented higher throughput than traditional PRNT, maintaining the correlation between results. Furthermore, when tested with dengue virus samples, it showed 50.53% less cross reactivity than MAC-ELISA. This fluorescent neutralization test could be used for clinical diagnosis confirmation of ZIKV infection, as well as for vaccine clinical trials and seroprevalence studies.

## Author summary

Since 2015, DENV’s cousin known as ZIKV has been in the spotlight. It caught researchers’ attention because it rapidly spread worldwide and ZIKV infection has been associated with Guillain-Barré syndrome cases and congenital brain abnormalities in newborn infants. For being so closely related, differentiation between DENV or ZIKV infection is challenging. Among the assays used in viral serological diagnosis, the plaque-reduction neutralization test (PRNT) that was described in the 1950s seems to be more specific, although longstanding, very laborious and not capable to test large number of samples. Therefore, we developed an image based neutralization test for ZIKV that overcomes restrictions presented by PRNT. This new test is faster, robust and able to test many samples simultaneously. It was successful in distinguish ZIKV infection from other infections, such as dengue and yellow fever. This may be especially relevant to solve cases such as congenital disorders in newborns and also to elucidate the agents involved in neuropathological outcomes such as Guillain-Barré syndrome. It also can be useful in serological surveys and vaccine studies.

## Introduction

Zika virus (ZIKV) is a mosquito-borne flavivirus that belongs to the Flaviviridae family, and is closely related to dengue virus (DENV). Flavivirus virions present a positive single-stranded RNA genome of approximately 11 Kb with a single open reading frame that encodes one polyprotein, which is further cleaved in 3 structural (C, prM and E) and 7 non-structural proteins (NS1, NS2A, NS2B, NS3, NS4A, NS4B and NS5) [1].

ZIKV was first isolated from a sentinel monkey in Uganda in 1947 [2] and, until 2007, it was considered endemic to Africa and Asia, when a small epidemic was reported in Yap State, Federated States of Micronesia [3]. In 2013, another ZIKV outbreak was reported in French Polynesia [4]. In 2015, ZIKV emerged in Brazil, and rapidly spread. By 2017, 48 countries and territories in the Americas had confirmed autochthonous ZIKV transmission [5–7].

In previous outbreaks, the illness was characterized by rash, conjunctivitis, subjective fever, arthralgia, and arthritis; infection appeared relatively mild, self-limiting, and nonlethal [3]. However, in recent outbreaks, an association with Guillain-Barré syndrome and congenital brain abnormalities in newborn infants of mothers infected with ZIKV during pregnancy has been observed [6,8,9]. These evidences indicate that an unequivocal diagnosis of the illness is of utmost importance for correct clinical management, especially in the case of pregnant women.

ZIKV diagnosis is based on clinical, epidemiological and laboratorial criteria. When samples are collected up to 5–7 days after the onset of symptoms, viral RNA can often be identified in serum or urine, and RT-PCR is the preferred test for ZIKV, and also for DENV and chikungunya virus (CHIKV) detection [10]. After this period, IgM antibodies may be detected by ELISA; however, flaviviruses have strong cross-reactivity, which may generate false positive results in serological tests [4,11]. This makes diagnosis of ZIKV infections quite a challenge, especially because the disease emerged in regions where other flaviviruses are endemic. Therefore, plaque-reduction neutralization test (PRNT) is indicated to measure virus-specific neutralizing antibodies and may be able to determine the etiology of infection [12].

Classical virus PRNT was first described in the 1950s and is considered the gold standard to measure neutralizing antibodies against viruses. Although being more specific, it is laborious and therefore not readily amenable to high-throughput, making it difficult to use for large-scale surveillance and vaccine trials.

In this study, we describe a fast and robust test to measure neutralizing antibody against ZIKV, which is suitable for high-throughput screening of large collections of serum specimens. This new assay is based on quantitative immunofluorescence, allying the classical PRNT format with a modern readout method.

## Methods

### Cells and virus

C6/36 *Aedes albopictus* cells (ATCC CRL-1660) were grown in Leibovitz L-15 medium (Gibco/Invitrogen, Grand Island, NY, USA) supplemented with 5% fetal bovine serum (FBS) (Gibco/Invitrogen, Grand Island, NY, USA), 0.26% tryptose (Sigma-Aldrich, St. Louis, MO, USA) and 25 μg/mL gentamicin (Gibco/Invitrogen, Grand Island, NY, USA) at 28°C. Human-derived hepatoma cells (Huh 7.5, ATCC PTA-8561) were grown in Dulbecco’s Modified Eagle Medium: Nutrient Mixture F-12 (DMEM/F-12 medium) (Gibco/Invitrogen, Grand Island, NY, USA) supplemented with 10% FBS and 100 IU/μg/ml penicillin/streptomycin (Gibco/Invitrogen, Grand Island, NY, USA) at 37°C in a humidified, 5% CO_2_-controlled atmosphere. D1-4G2-4-15 hybridoma was cultivated in RPMI-1640 medium (Gibco/Invitrogen, Grand Island, NY, USA) with 25 mM HEPES and supplemented with 10% FBS, 1 mM sodium piruvate, 250 ng/ml amphotericin B and 100 IU/μg/ml penicillin/streptomycin. ZIKV strain ZV BR 2015/15261 was isolated from a patient with zika fever from Northeast Brazil in 2015. Dengue viruses from four serotypes were used. DENV1- FGA/89 was isolated from a South American patient with dengue fever in 1989 (GenBank: AF226687). DENV2- ICC 265 and DENV3- BR DEN 97–04 (GenBank: EF629367) were isolated in Brazil. DENV4- LRV13/422 (GenBank: KU513441) was isolated from a non-fatal case of dengue with hemorrhagic manifestation. To obtain viral stocks, virus were propagated in C6/36 at the multiplicity of infection (MOI) of 0.01 and titrated by focus forming assay in C6/36.

### Serum specimens

A total of 226 sera were used in this study, which was approved by Fiocruz and the Brazilian National Ethics Committee of Human Experimentation (CAAE: 42481115.7.0000.5248), as well as the waiver of the Informed Consents. Specimens were divided as follows: 29 positive sera for ZIKV were confirmed by IgM ELISA and/or real time RT-PCR; 30 IgG sera positive for DENV confirmed with Panbio IgG indirect ELISA (Alere, Brisbane, Australia); 95 IgM sera positive for DENV (from all serotypes), confirmed by IgM capture ELISA and RT-PCR; 5 sera from yellow fever virus vaccinated volunteers; and 14 negative sera. Additionally, a panel of 53 samples positive for other acute infections was tested. This panel included sera positive for Toxoplasmosis (5 samples), Epstein-Barr virus (EBV) (10), Venereal Disease Research Laboratory test (VDRL) (17), Cytomegalovirus (CMV) (10), CMV/EBV (2), Leptospirosis (7), Hantavirus (2). With exception of Zika positive sera, all samples were collected prior to ZIKV emergence in Latin America.

Zika positive sera have been received in our laboratory since ZIKV outbreak in Brazil, when it was designated as a Sentinel Laboratory by the Brazilian Ministry of Health, thus working on ZIKV diagnosis in the South region.

### Plaque reduction neutralization test (PRNT)

Huh 7.5 cells were plated in 24 well plates at a density of 1x10^5^ cells, 16h previous to infection. Serum samples were inactivated at 56°C for 30 min, and then diluted 1/20 (followed by serial 1/3 dilutions). An equal volume of virus suspension containing 210 plaque-forming units (pfu) was mixed with diluted samples and incubated at room temperature for 1h. After this step, each mixture was inoculated onto plates with cells and after incubating at 37°C for 1h; inoculum was discarded and an overlay (1.6% CMC and 10% FBS in DMEM/F-12 medium) was added. Plates were left at 37°C for 6 days and then, cells were fixed with 3% paraformaldehyde and stained with 0.75% crystal violet. Plaques were counted and antibody titer was determined as the serum dilution that inhibited 90% of the tested virus inoculum (PRNT_90_).

### Fluorescent neutralization test

Huh 7.5 cells were plated in 96 well plates at a density of 1.5x10^4^ cells, 16h previous to infection. Serum samples were inactivated at 56°C for 30 min, and then diluted as described above. An equal volume of virus suspension (MOI of 0.4–300 pfu) was mixed with diluted samples and incubated at room temperature for 1h. Then, each mixture was inoculated onto plates with cells and incubated at 37°C for 1h. Inoculum was replaced with fresh medium and plates further incubated at 37°C for 48h.

Cells were fixed with cold methanol/acetone (v/v) and immunostained. Monoclonal antibody 4G2 (1/100) was used to stain virus envelope protein. It was diluted in blocking buffer (PBS with 1% BSA) and incubated at 37°C for 1h. Wells were washed three times with washing buffer (PBS with 0.05% tween 20) and incubated with secondary antibody anti-mouse IgG Alexa Fluor 488 (1/400) (Molecular Probes) in blocking buffer. Cell nuclei were counterstained with 5 μM DRAQ5 (Thermo Fisher Scientific) and washed three times with washing buffer.

Images were obtained with the Operetta High-Content Imaging System (PerkinElmer) with the objective 10x long WD. The number of images necessary to be representative for the entire well was defined and analyzed with Harmony High-Content Imaging and Analysis Software (PerkinElmer) (S1 Fig). Percentage of infected cells were obtained and normalized in relation to positive and negative controls; antibody titer was determined as the serum dilution that inhibited 90% of viral infection (NT_90_).

### Additional comparative methods

Zika IgM antibody capture enzyme-linked immunosorbent assay (MAC-ELISA) was performed accordingly to the guidelines from CDC [13] with minor modifications. A humanized monoclonal antibody (mAb) anti-flavivirus kindly provided by CDC was used as positive control. Antigens (ZIKV or Mock) were derived from β-propiolactone inactivated cell-culture supernatant from non-infected and ZIKV infected cells.

For ZIKV genome detection, viral RNA was extracted from 140 μL of samples using QIAamp viral RNA mini kit (Qiagen, Hilden, Germany). Real-time RT-PCR was performed as described by Lanciotti *et al*. (2008) [14], using 5 μL of RNA and Go-Taq Probe 1-Step RT-qPCR System (Promega). Assays were performed in the LightCycler 96 instrument (Roche, Mannheim, Germany) and human RNAse P was used as endogenous control [15].

### Statistical analysis

Assay quality was assessed by Z’ = 1– [3(σp+σn)/(μp- μn)], where σ is the standard deviation, μ is the mean of both positive (p) and negative (n) controls. Results were considered when Z’ was higher than 0.5 [16]. Neutralization curves were obtained using the software Prism (GraphPad version 6, USA) and PRNT_90_ and NT_90_ were calculated by the log (agonist) vs. response–Find ECanything curve, with a hillslope of 1.

## Results

### Fluorescent neutralization test development

To develop and validate the newly proposed fluorescent neutralization test as a potential substitute to the low throughput and labor intensive classical PRNT, we tested several parameters seeking for reproducible and faster results.

**Fig 1:**
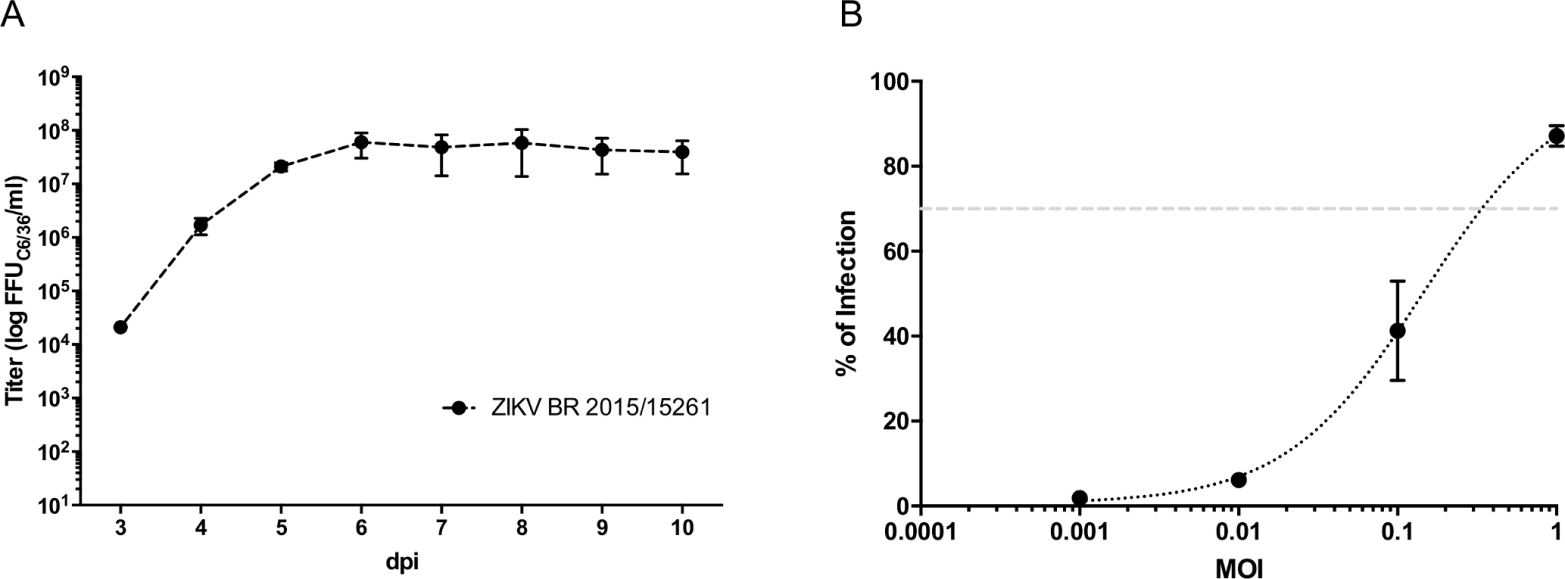
Infection parameters definition. Virus growth kinetics in C6/36 cell line (A). Zika virus infection in Huh 7.5 cell line with different MOIs (ranging from 0.001 to 1). Dashed line indicates 70% of infection (B). The data presented are the average results of three independent experiments.

ZIKV strain ZV BR 2015/15261 was chosen because it is a recent Brazilian clinical isolate and therefore a good representative to test serum samples from this region. Viral stocks were obtained from the second viral passage and by using low multiplicity of infection (MOI of 0.01) in C6/36 cell line, due to its good infection rates and low cytotoxicity. A kinetic of virus growth was performed between the third and tenth day after infection (Fig 1A) to determine the time point to recover culture supernatants. Viral stocks were harvested at the fifth day after infection during the middle to end of the exponential phases of growth, to avoid high concentrations of defective interfering particles that could lead to falsely low neutralization titers.

Huh 7.5, a human-derived hepatoma cell line, was chosen for the neutralization assays, because it is permissive to ZIKV and other flavivirus infection and also can be automatically well segmented with a software tool. The appropriate cell seeding density was defined as 1.5x10^4^ cells per well (34 mm^2^), since it has a sufficiently high number of cells but with enough spatial distribution for proper identification and accurate analysis. A MOI of 0.4 was used for all experiments because this condition yielded around 70% of infected cells after 48h (Fig 1B). Cell infection was visualized by an indirect immunofluorescence assay, with detection of ZIKV E protein by the 4G2 mAb and secondary anti-mouse IgG Alexa Fluor 488; nuclei were counterstained with DRAQ5. Four images per well (representative of the whole well) were acquired with the Operetta High-Content Imaging System and analyzed with the Harmony Analysis Software (PerkinElmer).

**Fig 2:**
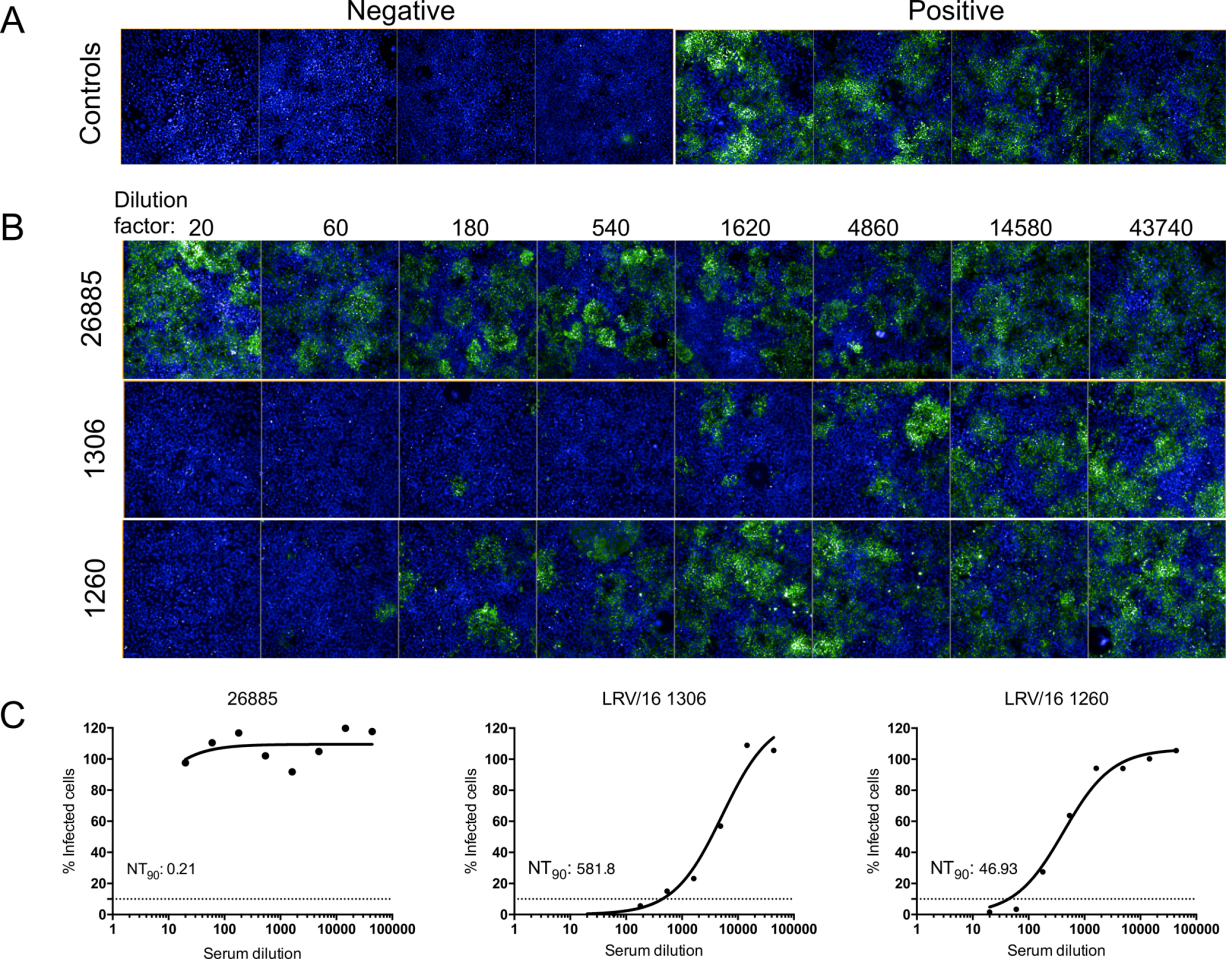
Fluorescent neutralization assay setting definition. Representative image of negative and positive infection controls (A). Assay overview of serial dilution (1/20 to 1/43740) of a negative sample (26885) and two positive samples (LRV/16 1306 and LRV/16 1260) (B). Curve fitting of results and calculation of neutralization titer that inhibit 90% of viral infection (NT_90_) (C).

After the standardization step, we proceeded to the neutralization assay. Serum specimens were heat inactivated to reduce the effects that complement factors may have on final results. Serum and virus samples were mixed to allow neutralization. After the incubation period the mixture was added to cells so infection could occur by non-neutralized virus. The neutralization titer that inhibits 90% of viral infection (NT_90_) was used to analyze results (Fig 2).

Some criteria were followed in order to accept a valid assay. Among them, a uniform number of cells per well, appropriate percent of infection of controls, no serum toxicity observed with low serum dilutions, and a Z’ higher than 0.5.

In order to observe inter-assay variability, one negative and one positive sample were tested in three independent assays. It was observed a low variation for the negative (0.93 ± 0.16), and for the positive samples (188.46 ± 3.01), showing the robustness of the test. The average Z’ observed for all plates was 0.61.

### Comparison to PRNT

**Fig 3:**
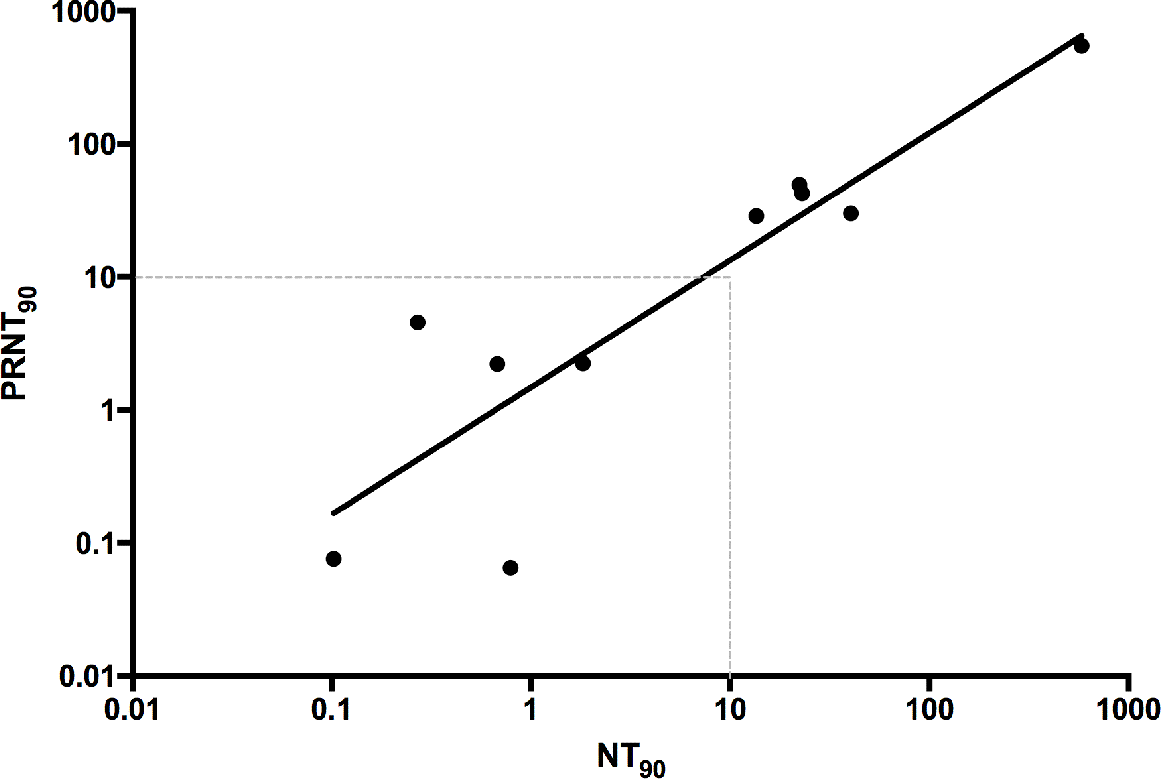
Neutralization tests comparison. Neutralization titers obtained from PRNT or fluorescent neutralization test are compared. A correlation of R = 0.88 was obtained (P = 0.0003).

PRNT is the gold standard for measurement of flavivirus neutralization. Therefore, neutralizing titers obtained from 12 serum samples by using either the new fluorescent neutralization test and or classical PRNT were compared. Similar neutralization results were obtained with the two approaches, with a correlation of 0.88 (Fig 3). This demonstrates the robustness of the newly developed test and that it could be used as a replacement of the traditional test, using the same interpretation guidance suggested by CDC [17].

### Fluorescent neutralization test validation

**Fig 4:**
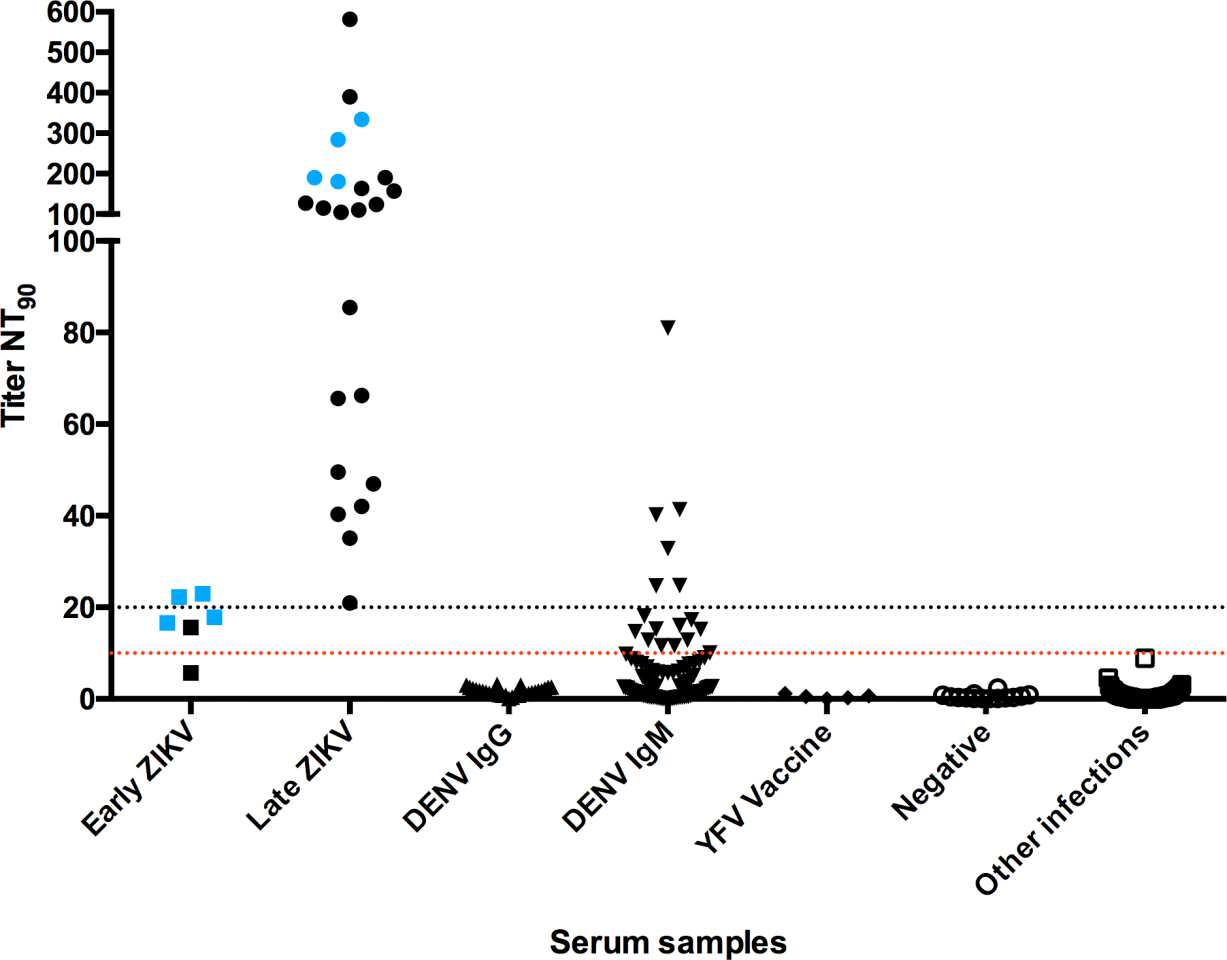
ZIKV Fluorescent neutralization test validation. A total of 226 serum samples, among then negative and positive for flavivirus and non-flavivirus infections were examined. The neutralization titers that inhibit 90% of viral infection (NT_90_) are shown in the graph. Other infections: Leptospirosis, CMV, EBV, Hantavirus, Toxoplasmosis, VDRL. Dashed lines represent the test cut off: negative when NT_90_ <10, inconclusive when NT_90_ ≥10 and <20, and positive when NT_90_ ≥20. Symbols in blue indicate paired samples.

The new proposed test was validated with a set of serum samples previously tested. This panel included sera positive for flavivirus and non-flavivirus acute infections and negative serum from healthy donors (Table 1). Zika positive samples were collected during the disease outbreak in Brazil; all the other samples were collected previous to the ZIKV emergence in the country. All samples were submitted to the fluorescent neutralization test and the NT_90_ was calculated (Fig 4).

**Table 1:** Panel of serum samples used for the new ZIKV fluorescent neutralization test validation.

Serum Samples	n
Zika virus	29
Dengue virus	125
Yellow fever vaccine	5
Leptospirosis	7
Cytomegalovirus (CMV)	10
Epstein- Barr virus (EBV)	10
CMV/ EBV	2
Hantavirus	2
Toxoplasmosis	5
VDRL positive	17
Negative	14
Total	226

Following recommended interpretation for neutralization results [17], a titer higher than 10 is supposed to be considered positive. However, it was observed that several IgM positive samples for dengue would be erroneous considered positive for ZIKV. Therefore, a more restrictive result interpretation was employed as follows: samples were considered negative when NT_90_ <10, inconclusive when NT_90_ ≥10 and <20, and positive when NT_90_ ≥20.

Even with the higher cutoff value, it was possible to observe six DENV IgM samples that cross-reacted in ZIKV neutralization assay; while another 10 samples were inconclusive. No cross reactivity was observed when samples of DENV IgG, other acute infections or YFV vaccine were analyzed.

Regarding the Zika positive panel (Table 2), samples were tested by Zika MAC-ELISA and/ or real time RT-PCR, and then divided into two groups: early infection (serum PCR positive and variable anti-ZIKV IgM) and late infection (PCR negative and anti-ZIKV IgM positive samples). PCR positive samples presented low neutralizing titers (<23), while NT_90_ of PCR negative /IgM positive samples ranged from 20.98 to 581.80.

**Table 2:** Characterization of Zika positive serum samples.

Sample	Zika MAC-ELISA	RT-PCR	NT_90_	Observation
397	Positive	ND^a^	85.48	ZIKV PCR+ paired sample^b^
399	Positive	ND	114.80	ZIKV PCR+ paired sample^b^
401	Positive	ND	126.70	ZIKV PCR+ paired sample^b^
403	Positive	ND	156.70	ZIKV PCR+ paired sample^b^
405	Positive	ND	104.10	ZIKV PCR+ paired sample^b^
407	Positive	ND	66.22	ZIKV PCR+ paired sample^b^
409	Positive	ND	65.63	ZIKV PCR+ paired sample^b^
411	Positive	ND	123.60	ZIKV PCR+ paired sample^b^
413	Positive	ND	49.56	ZIKV PCR+ paired sample^b^
415	Positive	ND	35.14	ZIKV PCR+ paired sample^b^
LRV/16 081	Positive	Negative	20.98	Urine and placenta ZIKV PCR+
LRV/16 464	Positive	Negative	110.50	Urine ZIKV PCR+
LRV/16 529	Positive	Negative	42.04	Colostrum ZIKV PCR+
LRV/16 794	Positive	ND	190.40	DENV IgM negative
LRV/16 1034	Positive	Negative	284.40	ZIKV PCR+ paired sample (LRV/16 1257)
LRV/16 1044	Positive	Negative	190.00	ZIKV PCR+ paired sample (LRV/16 1249)
LRV/16 1058	Positive	Negative	334.20	ZIKV PCR+ paired sample (LRV/16 1263)
LRV/16 1074	Inconclusive	Negative	180.50	ZIKV PCR+ paired sample (LRV/16 1253)
LRV/16 1243	Positive	Negative	163.60	DENV IgM negative
LRV/16 1255	Positive	Negative	40.32	DENV IgM negative
LRV/16 1260	Positive	Negative	46.93	DENV IgM negative
LRV/16 1268	Positive	Negative	390.40	DENV IgM negative
LRV/16 1306	Positive	Negative	581.80	DENV IgM negative
LRV/16 022	Negative	Positive	5.67	
LRV/16 1249	Positive	Positive	17.81	
LRV/16 1253	Positive	Positive	22.23	
LRV/16 1257	Positive	Positive	22.96	
LRV/16 1262	Positive	Positive	15.61	
LRV/16 1263	Negative	Positive	16.62	

^a^ND: Not done

^b^IOC Panel: ZIKV IgM positive serum panel provided by the Oswaldo Cruz Institute (Fiocruz). These sera have a paired sample with a ZIKV PCR positive result.

**Fig 5:**
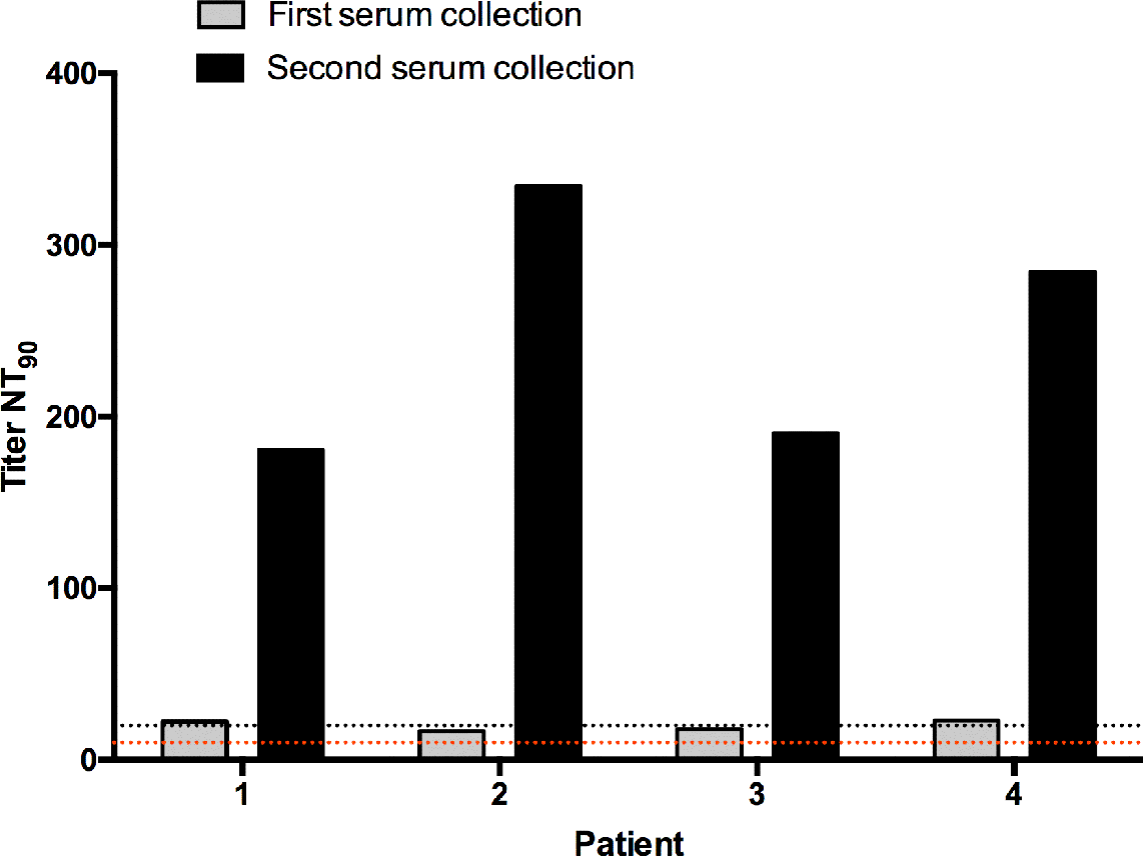
ZIKV neutralization test with paired samples. Second collections were performed from 3 to 6 months after the first one. Dashed lines represent test cut off: negative when NT_90_ <10, inconclusive when NT_90_ ≥10 and <20, and positive when NT_90_ ≥20.

Paired samples (presented in blue in Fig 4) were obtained from four patients; first collections were all RT-PCR positive and presented low NT_90_ titers, while second collections obtained 3 to 6 months after the first one had neutralization titers increased to levels a lot higher than the cut off value (Fig 5).

### Cross reactivity with DENV IgM positive samples

To assess the cross reactivity between ZIKV and DENV infections in MAC-ELISA format assay and the fluorescent neutralization test, 95 DENV well-characterized positive samples were tested (Table 3). Neutralization titers of these samples are presented in Fig 4. This sample panel was obtained between the years of 2004 and 2006 in Venezuela, thus before the emergence of ZIKV in the region. It is worth mentioning that the panel is composed by paired samples and viral isolation during acute phase of infection was used as “gold standard” for DENV infection.

**Table 3:** Panel of DENV IgM positive samples.

Serotype	n	%
DV1	47	49.47
DV2	6	6.32
DV3	37	38.95
DV4	2	2.10
N^a^	3	3.16
Total	95	100.00

^a^ Sample not serotyped

Among the 95 IgM DENV positive samples tested by MAC-ELISA, 39 cross-reacted and presented false positive results for ZIKV and 25 were inconclusive or undetermined. On the other hand, only six samples presented false positive results for ZIKV and 10 were inconclusive in the neutralization test (Table 4). Thus, the novel neutralization test presented 50.53% less cross reactivity than MAC-ELISA, and the rate of correct identification of ZIKV negative serum increased from 32.63% to 83.16%.

**Table 4:** Comparison of Zika MAC-ELISA and the new ZIKV fluorescent neutralization test with IgM DENV positive samples.

ZIKV Fluorescent Neutralization Test	Zika MAC- ELISA			Total
	Negative	Inconclusive/ Undetermined	Positive	
Negative	31	20	28	79
Inconclusive	0	1	9	10
Positive	0	4	2	6
Total	31	25	39	95

### Fluorescent neutralization test expansion

The fluorescent neutralization test format can be expanded to other diseases. As a proof of concept, the test was adapted to identify neutralization antibodies to dengue virus. The same standardization steps used previously were employed to develop a test for the four serotypes of DENV. Optimal harvest time for viral stocks was between the 6 and 7^th^ day after infection and, in order to obtain around 70% of infection after 48h, a MOI of 0.1 was used. DENV fluorescent neutralization test was able to identify neutralization antibodies against the four serotypes of the virus in all DENV IgM positive samples tested (Table 5). However, it was not possible to identify which DENV serotype was responsible for the current infection according to fluorescent neutralization assay results, indicating a probable secondary DENV infection.

**Table 5:** DENV fluorescent neutralization test results for sera of patients with proven DENV infection.

Serum Sample	DENV Serotype	DENV Fluorescent Neutralization Test (NT_90_)			
		DENV-1	DENV-2	DENV-3	DENV-4
27242	DV1	**467.80**	514.80	415.50	25.17
27402	DV1	**311.60**	733.20	604.10	74.83
23535	DV1	**25.33**	13.15	21.35	0.43
31284	DV2	2,745.00	**1,264.00**	4,189.00	759.60
31462	DV2	3,559.00	**1,099.00**	220.80	48.92
22464	DV3	700.90	658.20	**466.00**	59.21
22456	DV3	3,365.00	1,411.00	**2,600.00**	633.90
22510	DV3	318.30	773.40	**436.40**	240.50
23447	DV4	76.13	66.25	94.72	**48.04**
1757	Negative	2.65	5.62	1.88	1.70
2033	Negative	3.15	6.75	0.43	0.01

## Discussion

Since ZIKV emerged in South America causing a number of outbreaks with reported cases associated with Guillain-Barré syndrome and congenital brain abnormalities in newborn infants, a great effort to develop specific and reliable diagnosis tests has been made [18–20].

A definitive ZIKV diagnosis is achieved by detecting viral RNA in patient serum, or other samples like urine, semen and placenta. Although RT-PCR assay is trustworthy and with good sensitivity and specificity, viremia among ZIKV-infected patients are relatively low and detectable for only a few days after the onset of symptoms [14].

For individuals beyond this viremia window, a serologic test must be employed. The most common used method is the detection of reactive IgM antibodies by ELISA. A disadvantage in using this option for ZIKV is the high number of false positive results due to cross-reaction with antibodies against DENV, and the low sensitivity of most existing immunoassays [11,21]. A novel ELISA based on recombinant ZIKV non-structural protein 1 (NS1) was able to eliminate cross-reactions with antibodies to DENV and other flaviviruses, although it presented low sensitivity in the IgM format [18].

In order to overcome this issue, a CDC diagnostic guideline recommends that presumptive positive or equivocal MAC-ELISA result for ZIKV needs to be verified with a confirmatory PRNT [10].

As previous stated, although PRNT is the gold standard for flavivirus serological test, a number of limitations prevents its use in large scale to test a great number of samples, as required during outbreaks or to perform serological surveys. In addition, there is a recommendation for serological testing of asymptomatic pregnant women with history of travel to ZIKV endemic regions or those living in areas with active viral transmission [22].

This study describes the development and validation of a novel image based neutralization test for ZIKV that overcomes restrictions presented by PRNT. Previous studies have developed assays for replacement of DENV PRNT. Vorndam and Beltran (2002) developed and evaluated a microneutralization test to measure anti-dengue antibodies using an *in situ* ELISA [23]. Additionally, a 96-well format flow cytometry-based neutralization assay was proposed, and similar neutralization patterns were observed when compared to classical PRNT [24]. The disadvantages observed included the high intra-assay variability and the need to remove adherent cells from wells.

Recently some alternative assays to ZIKV PRNT have also been reported. A MTT-based cell viability assay for ZIKV neutralizing antibodies quantification has been developed, and although it does not require expensive equipment or costly reagents, it depends on virus-induced cytopathic effect [25]. Shan *et al*. (2017) developed a reporter virus neutralization test (RVNT), based on ZIKV and DENV luciferase reporter viruses. The assay maintained relative specificity of traditional PRNT and was further evaluated with 258 clinical serum specimens, displaying a 93.1% agreement with the traditional ZIKV PRNT titers [26,27]. Furthermore, a neutralization assay in which the endpoint is measured by real-time PCR was proposed [28].

The novel fluorescent neutralization assay developed here combines the classical neutralization protocol with a new automatized readout method, employing a high-content imaging system. From seeding cells to obtaining results, the new test takes around 72h, in contrast to PRNT that can take up to 8 days [29], and also depends on manual counting of plaques, which can vary from person to person. Besides that, this new assay is able to test at least ten serum samples against a virus on a single 96-well plate, with dilutions performed via multichannel pipetting devices that increase assay capacity.

Maistriau *et al*. (2017) also proposed a fluorescent neutralization test using a high-throughput image acquisition system. However, it is based on the translocation of the transcription factor IRF3 in response to infection [30], thus requiring a careful selection of cell lines according to the virus of interest. In contrast, we propose a robust and simple method that can be easily set up to investigate other flavivirus infections.

In this study, a curve fitting method from several serum dilutions was used to calculate neutralization titers, which allows a more precise result, in contrast to simply report the reciprocal of the last serum dilution that shows 50 or 90% reduction of infection. The neutralization titer which inhibits 90% of viral infection (NT_90_) was used because it is indicated for epidemiological studies or diagnostic purposes in endemic areas, decreasing background serum cross-reactivity among flaviviruses [29].

It has been reported that people exposed to secondary DENV infections develop broadly neutralizing antibodies that neutralize different serotypes other than the one responsible for current infection [31], as it was also demonstrated in the DENV fluorescent neutralization test (Table 5). Additionally, sera from patients with secondary DENV infection exhibit potent cross-reactivity against ZIKV [11]. In this context, cross reactivity between ZIKV and DENV is quite expected, as the viruses envelope proteins share a high degree of homology with a sequence identity of 54% and nearly identical structures. The fusion loop, that is an important antibody target, is 100% conserved between the two viruses [11]. Therefore, increasing specificity of serological tests is particularly relevant, since ZIKV emerged in flavivirus endemic regions.

Aiming to reduce false positives results, samples were considered ZIKV positive when NT_90_ ≥20, while NT_90_ <10 samples were scored as negatives. When NT_90_ ranged from ≥10 to <20, results were recorded as inconclusive. Other studies have also used a higher cut off PRNT_90_ value [3,28]. Based on those parameters, higher specificity was achieved when compared to MAC-ELISA, yielding in less ZIKV false positive results for DENV IgM positive serum samples. Only 10.53% of inconclusive and 6.32% of false positive results were observed with these settings.

This result is particularly remarkable, since in another assay, up to 100% of cross reaction with ZIKV was observed when acute and convalescent sera from nine Thai patients with confirmed DENV infection by RT-PCR were tested, both in binding and in neutralization assays [11]. Another study using RVNT for anti-ZIKV antibodies detection, showed 20% of erroneous results in the presence of anti-DENV antibodies, although no false positive results with Yellow fever and West Nile positive samples were observed [27]. The real-time PCR neutralization assay also reported significant cross-reactivity when testing a serum specimen from a patient with proven current ZIKV infection which had a background of DENV infection [28].

It is noteworthy that higher cut off values may reduce assay sensibility, i.e, some samples of ZIKV early infections can become inconclusive. In those cases, a molecular diagnosis can be employed and/ or a second serum collection should be tested, since antibodies might not have yet reached detectable levels. This was observed when paired samples were tested and an increase in neutralization titers was observed.

As a conclusion, the developed fluorescent neutralization test offers significant advantages over classical PRNT. It is faster, prompt to high throughput adaptation, has automated reading of results, and is more specific than MAC-ELISA assay. As expected it also presents some limitations, as it does not discriminate between antibody classes, requires expensive equipment and can be performed only in selected laboratories. Nevertheless, it will make it possible to test simultaneously a large number of samples and against different viruses, assisting the correct management of suspected patients or asymptomatic pregnant woman and be employed in seroprevalence surveys.

## Supporting information

S1 Fig Harmony high-content imaging analysis. Using the input image, cell nuclei and cytoplasm were identified. The intensity of green fluorescence was calculated and a population selected. The values were transferred to a table and the neutralizing titers calculated.
(TIF)
