# Supplementary figures and images for: Development and evaluation of a novel high-throughput image-based fluorescent neutralization test for detection of Zika virus infection
Source: PLoS Negl Trop Dis. 2018 Mar 15;12(3):e0006342. doi: 10.1371/journal.pntd.0006342 (PMC5871014; doi:10.1371/journal.pntd.0006342)

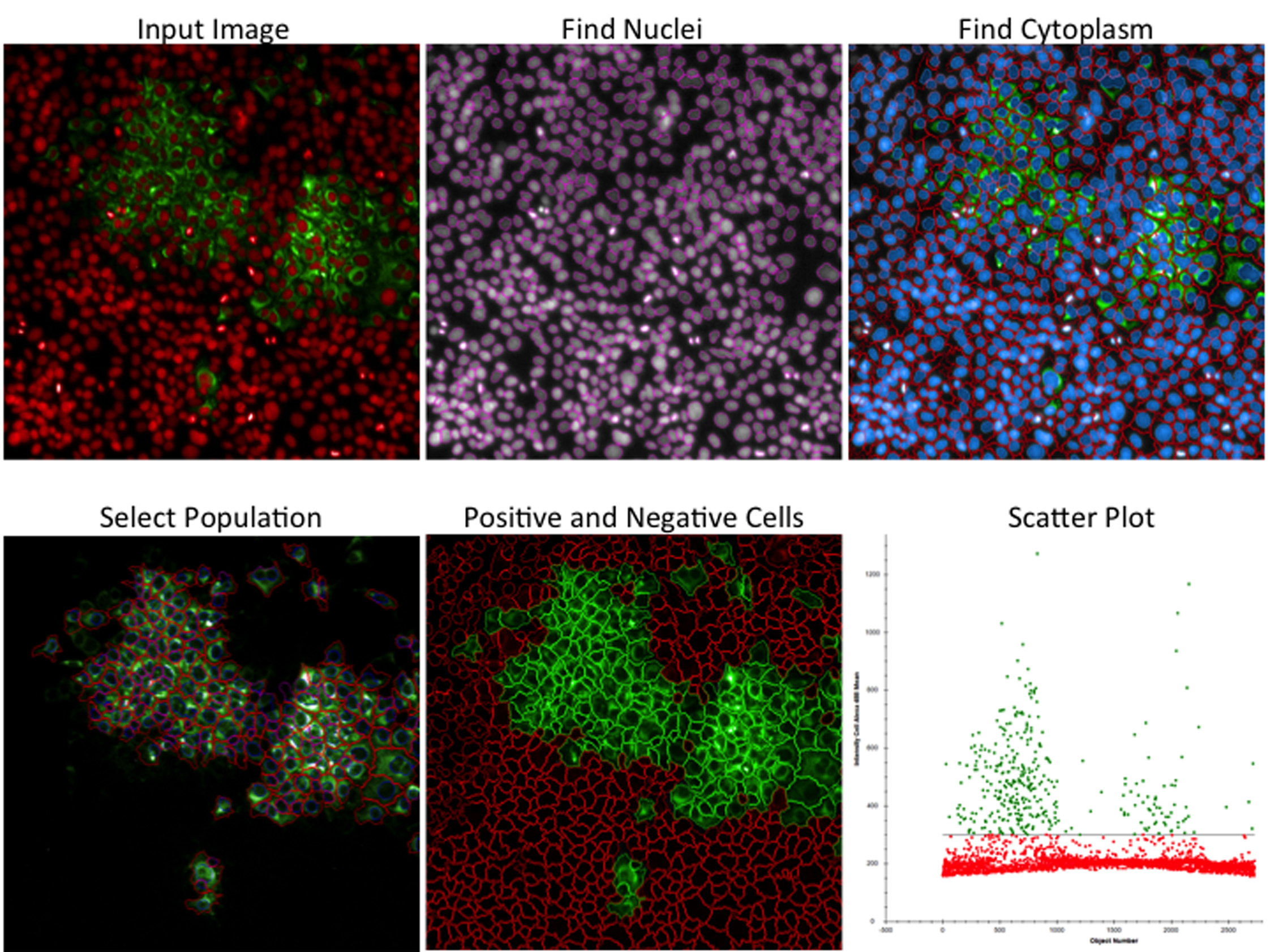

Supplement: S1 Fig — Using the input image, cell nuclei and cytoplasm were identified. The intensity of green fluorescence was calculated and a population selected. The values were transferred to a table and the neutralizing titers calculated. (TIF) [file pntd.0006342.s001.tif]
